# Bridging Oxidation and Crystallization Pathways in Sn–Pb Perovskites for High‐Efficiency, Stable Solar Cells

**DOI:** 10.1002/cssc.202502028

**Published:** 2026-01-27

**Authors:** Manman Hu, Jens Hauch, Jianchang Wu, Christoph Brabec

**Affiliations:** ^1^ Forschungszentrum Jülich GmbH HelmholtzInstitute Erlangen−Nürnberg (HI‐ERN) Erlangen Germany; ^2^ Faculty of Engineering Department of Material Science Materials for Electronics and Energy Technology (i‐MEET) Friedrich‐Alexander‐Universität Erlangen−Nürnberg (FAU) Erlangen Germany; ^3^ Forschungszentrum Jülich GmbH Institute of Energy Materials and Devices (IMD) Jülich Germany

**Keywords:** device stability, Pb–Sn perovskite, perovskite, precursor engineering, tandem solar cells

## Abstract

All‐perovskite tandem solar cells (TSCs) have recently surpassed the 30% power conversion efficiency milestone, positioning mixed tin–lead (Sn–Pb) perovskite as indispensable narrow‐bandgap absorbers. Their optimal bandgap, reduced lead content, and solution processability make them promising for next‐generation photovoltaics. However, their widespread application is hindered by severe stability issues, primarily the facile oxidation of Sn^2+^ and crystallization mismatch between Sn‐ and Pb‐based phases. Distinct from existing reviews, this short review provides an integrated framework for the two fundamental bottlenecks of Sn–Pb perovskite—Sn^2+^ oxidation and Sn/Pb crystallization mismatch—linking mechanistic insights across precursor chemistry, thin‐film formation, and device operation. We summarize recent advances that enable efficiencies >23% together with thousand‐hour operational stability, and we outline future directions toward fully integrated, scalable, and commercialization‐relevant stability solutions.

## Graphical abstract

This review summarizes recent progress in stabilizing mixed Sn–Pb perovskite solar cells by bridging two fundamental bottlenecks: Sn^2+^ oxidation and Sn/Pb crystallization mismatch. We highlight precursor redox control, molecular antioxidants, vacancy suppression, and interfacial engineering strategies that jointly regulate oxidation chemistry and crystallization kinetics, enabling high efficiency (>23%) and long‐term operational stability for narrow‐bandgap perovskite solar cells and all‐perovskite tandems.

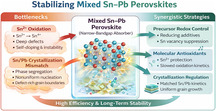

## 1 Introduction

All‐perovskite tandem solar cells (TSCs) have emerged as one of the most competitive candidates for next‐generation photovoltaics, with certified power conversion efficiencies (PCEs) exceeding 30% in recent years. This impressive performance stems from the ability to combine a wide‐bandgap (WBG) top cell (≈1.7–1.9 eV) with a narrow‐bandgap (NBG) bottom cell (≈1.2–1.3 eV), enabling optimal spectral utilization. Among NBG absorbers, mixed tin–lead (Sn–Pb) halide perovskite solar cells (PSCs) are particularly attractive due to their tunable bandgap via compositional engineering and their compatibility with low‐temperature solution processing.

The strong bandgap bowing effect in Sn–Pb alloys allows the bottom cell to achieve the optimal ≈1.2 eV bandgap for tandem current matching. For example, integrating high‐quality Sn–Pb subcells with WBG counterparts has yielded tandem efficiencies above 30%, approaching the practical commercial threshold [1]. Furthermore, the use of Sn reduces the overall lead content, addressing environmental concerns associated with pure Pb‐based perovskites. However, despite these advantages, the practical application of Sn–Pb perovskites is hampered by intrinsic chemical and structural instabilities, as well as environmental degradation pathways: (1) Chemical instability: Sn^2+^ is highly susceptible to oxidation to Sn^4+^, even under mild conditions, introducing deep‐level defects and severely reducing device performance. (2) Crystallization mismatch: Sn‐ and Pb‐based perovskite phases exhibit different nucleation and growth rates, often leading to compositional gradients, rough surfaces, and high defect densities. (3) Environmental vulnerability: Exposure to oxygen, moisture, heat, and light can accelerate degradation through ion migration, defect formation, and phase reconstruction [2].

These two challenges are not only independent degradation routes but can also be mutually reinforcing: poor crystallization uniformity exposes more defect sites, which, in turn, accelerates oxidation. In this review, we analyze recent advances in overcoming Sn^2+^ oxidation and crystallization mismatch in Sn–Pb perovskites, summarizing strategies that enhance both optoelectronic quality and long‐term operational stability, and distilling universal design rules applicable to other perovskite systems, including lead‐free compositions.

## 2 Chemical Oxidation of Sn^2+^

Sn^2+^ oxidation is the most critical degradation pathway for Sn–Pb perovskites. This process is both thermodynamically favorable and kinetically facile, occurring during precursor storage, film formation, and device operation [3, 4]. Oxygen, moisture, and iodine vacancies (V_I_) are key accelerants of this reaction. The oxidation from Sn^2+^ to Sn^4+^ increases p‐type self‐doping, raises background carrier density, and introduces deep trap states, which shorten carrier lifetimes and increase nonradiative recombination losses [5, 6, 7, 8].

**FIGURE 1:**
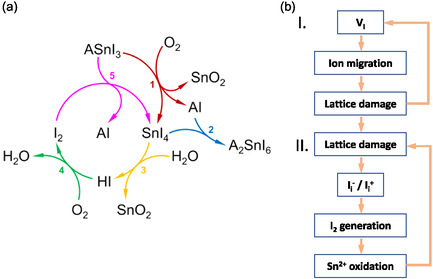
Schematic illustration of the Sn–Pb perovskite degradation pathways (a) light‐induced defect‐assisted oxidation under oxygen/moisture. Reproduced under terms of the CC‐BY license [9]. Copyright 2021, Springer Nature. “A” refers to the organic cations chosen for the preparation of the hybrid tin perovskite. (b) Under inert atmosphere. Reproduced with permission [10]. Copyright 2023, John Wiley and Sons.

Recent mechanistic studies on Sn‐based perovskites have revealed a self‐accelerating oxidation cycle that is also relevant for mixed Sn–Pb compositions [9]. Figure 1a illustrates the cyclic degradation pathway of tin–iodide perovskites under ambient air exposure. The process is initiated by the oxidation of the perovskite (ASnI_3_) by molecular oxygen to yield SnI_4_ (Reaction 1). SnI_4_ can subsequently react with the organic iodide component (AI, where A ≡ FA or PEA) to form the vacancy‐ordered phase A_2_SnI_6_ in the solid state (Reaction 2). In the presence of moisture, SnI_4_ undergoes hydrolysis to generate hydroiodic acid (HI) (Reaction 3), which is further oxidized by oxygen to produce molecular iodine (I_2_) and water (Reaction 4). The generated I_2_ is a potent oxidizing agent that can react with residual perovskite to regenerate SnI_4_ (Reaction 5), thus closing the degradation loop. This feedback mechanism accelerates structural decomposition and iodine loss, leading to rapid performance decay. Interrupting this cycle—such as by inhibiting O_2_ adsorption, suppressing SnI_4_ formation, or scavenging I_2_—is therefore crucial for improving the stability of Sn‐based perovskites.

**FIGURE 2:**
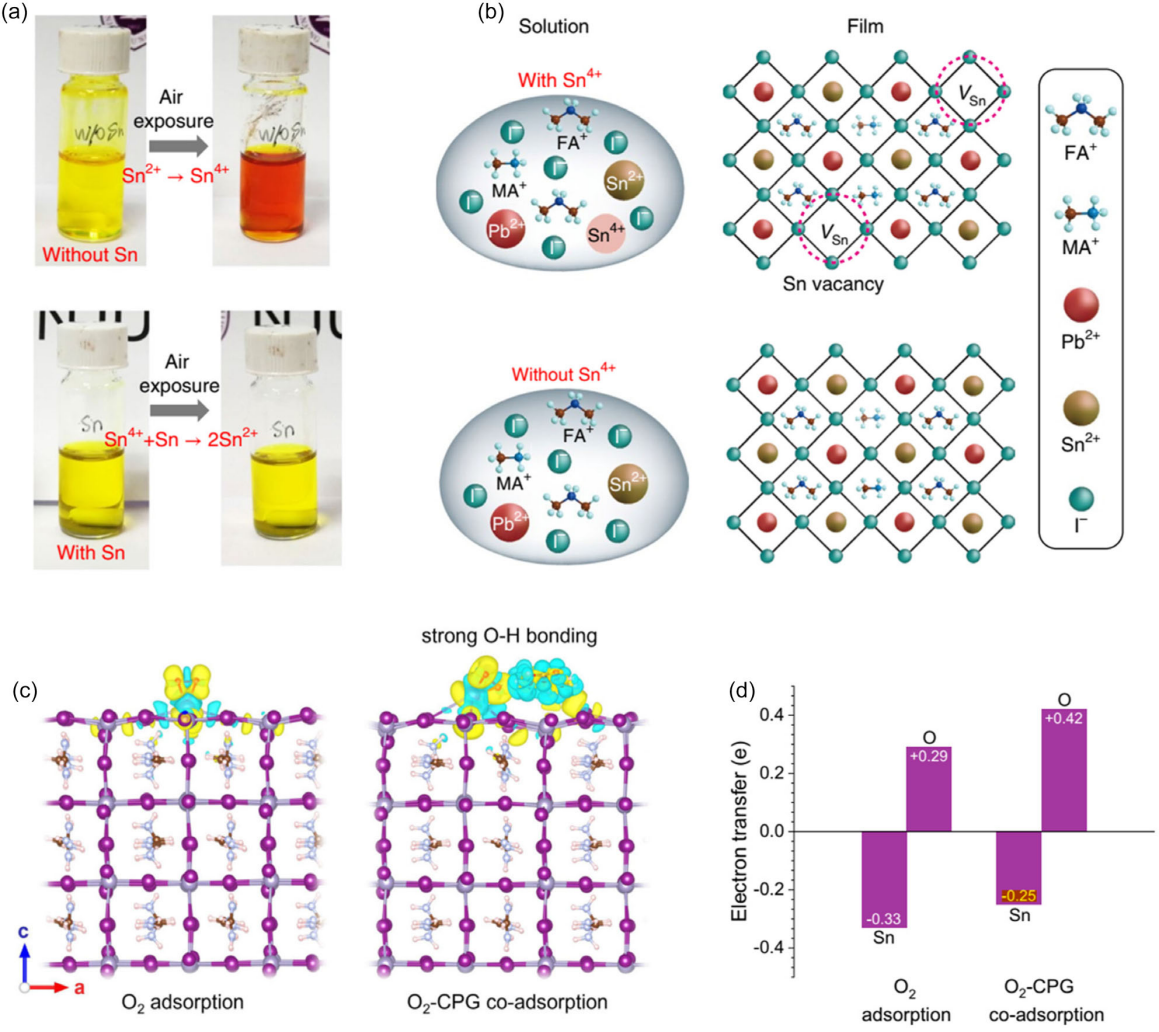
(a) Photographs showing the ease of oxidation of Sn^2+^ to Sn^4+^ in ambient air and the facile reduction of Sn^4+^ to Sn^2+^ by metallic Sn powders. (b) Illustration of the formation of Sn vacancies in mixed Pb–Sn perovskite due to the presence of Sn^4+^ in the precursor solution and the suppression of Sn vacancy formation in TRP perovskite because of the absence of Sn^4+^. Reproduced with permission [3]. Copyright 2019, Springer Nature. (c) Distributions of charge density induced by O_2_ adsorption and O_2_‐CPG coadsorption on the surfaces of Sn–Pb perovskites. (d) Electron transfer of O atoms in O_2_ and Sn ions in perovskites under O_2_ adsorption and O_2_‐CPG coadsorption. Reproduced under terms of the CC‐BY license [13]. Copyright 2024, Springer Nature.

**FIGURE 3:**
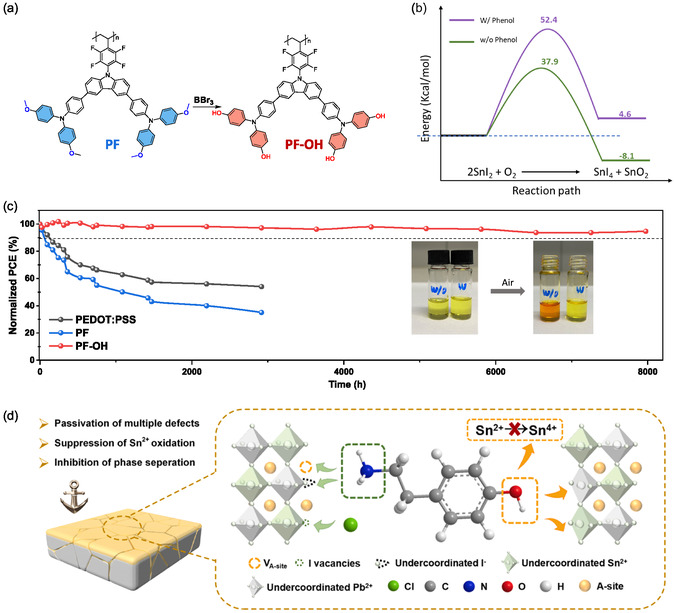
(a) Synthetic route of PF–OH. (b) SnI_2_ oxidation process and reaction energy barrier. (c) The long‐term stability of unencapsulated cells stored in an N^2^‐filled glovebox. The inserted photographs show the ease of oxidation of Sn^2+^ to Sn^4+^ in ambient air and the inhibition of this process by PF–OH. Reproduced under terms of the CC‐BY license [18]. Copyright 2025, John Wiley and Sons. (d) Schematic diagram of the passivation of various defects and the inhibition of Sn^2+^ oxidation in the perovskite thin films caused by OHPEACl. Reproduced with permission [19]. Copyright 2023, Elsevier.

Sn^2+^ oxidation originates as early as the precursor stage, where trace Sn^4+^ impurities or oxygen‐rich contaminants can trigger the formation of Sn vacancies*.* Zhu et al. [11] showed that the purity of SnI_2_ and organic salts directly determines the intrinsic defect density of the final perovskite film, highlighting the importance of rigorous precursor‐quality control. In addition to purity, the precursor redox environment plays a decisive role. Zhang et al. [12] demonstrated that metallic Pb powder can effectively reduce Sn^4+^ back to Sn^2+^ through a comproportionation reaction, stabilizing the precursor ink and suppressing vacancy formation. Complementing this chemistry‐based approach, Lin et al. report a simple and effective tin‐reduced precursor (TRP) solution strategy, where a small amount of metallic Sn powder is added into the precursor solution (Figure 2a,b) [3]. The metallic Sn acts as a sacrificial reductant to prevent Sn^2+^ from oxidizing into Sn^4+^ during precursor storage and processing. Excess Sn particles are removed by filtration before film deposition, ensuring film purity. This method reduces Sn vacancies within the perovskite grains, leading to an extended carrier diffusion length of up to 3 µm—comparable to that of high‐quality Pb‐based perovskites—and PCEs exceeding 22%. For 1.22 eV bandgap Sn–Pb PSCs, a maximum PCE of 21.1% was achieved. These results highlight the potential of precursor‐state reduction chemistry to simultaneously improve both the optoelectronic quality and operational stability of NBG Sn–Pb devices. These improvements have made TRP a widely adopted step in high‐performance Sn–Pb perovskite fabrication, and recent progress leveraging TRP has enabled state‐of‐the‐art device efficiencies exceeding 24.9% [4, 14, 15, 16, 17]. Oxygen adsorption is the initial step in this cycle. Zhou et al. demonstrated that introducing a multifunctional N‐(carboxyphenyl)guanidine hydrochloride (CPGCl) molecule, which forms hydrogen bonds with O_2_, results in O_2_‐CPG coadsorption and reduces oxygen binding on Pb–Sn perovskites (Figure 2c,d) [13]. Moreover, the strong coordination between CPGCl molecules and charged defects increases the local electron density at these sites, thereby creating substantial kinetic barriers to Sn^2+^ oxidation. CPGCl modification enables mixed Sn–Pb PSCs to achieve high efficiency and excellent stability, with single‐junction devices reaching a stabilized PCE of 23.11% and retaining 97.45% of their initial performance after 3500 h in inert storage, while tandem devices deliver a certified PCE of 27.35%. Similarly, Wu et al*.* showed that phenol‐functionalized HTLs strongly coordinate with Sn^2+^, suppressing oxygen adsorption on Sn^2+^ sites (Figure 3a‐c). Phenol‐functionalized PF–OH (poly‐4,4′,4″,4′″‐(((9‐(2,3,5,6‐tetrafluoro‐4‐vinylphenyl)‐9*H*‐carbazole‐3,6‐diyl)bis(4,1‐phenylene))bis(azanetriyl))tetraphenol) was found to effectively inhibit oxidation of Pb–Sn perovskite precursor solutions: control solutions exposed to air rapidly changed from light yellow to orange‐red, whereas PF–OH‐containing solutions retained their color for extended periods. DFT calculations revealed that PF–O^–^ increases the activation energy of the oxidation reaction, accounting for its inhibition effect. As a result, the shelf stability of the devices increased by around 100 times. Analogous results were reported by Tian et al., who found that ammonium salts bearing phenolic hydroxyl groups (4‐hydroxyphenethylamine hydrochloride, OHPEACl) also suppress oxidation in Pb–Sn perovskites (Figure 3d) [19]. In practical terms, breaking this cycle requires multipronged strategies: suppressing SnI_4_ formation, removing or scavenging I_2_, reducing V_I_ density, and limiting oxygen/moisture ingress. Molecular antioxidants, vacancy fillers, and dense encapsulation layers are thus key to long‐term operational stability.

Sn–Pb PSCs degrade much faster under continuous illumination than their Pb‐based counterparts, even when stored under inert atmospheres or fully encapsulated to exclude moisture and oxygen [20, 21, 22, 23]. Even after film formation, Sn^2+^ oxidation continues during device operation and is strongly accelerated under illumination. Sn–Pb PSCs degrade faster than their Pb‐based counterparts even under inert or encapsulated conditions, indicating that external oxygen and moisture are not the only driving forces. Yun et al. [24] revealed that accumulated photogenerated holes at the perovskite/HTL interface can directly oxidize Sn^2+^ to Sn^4+^, introducing lattice distortion and triggering long‐term instability. In parallel, illumination induces iodide‐related photochemical processes, including the formation of iodine vacancies (V_i_) and interstitial iodides (I_i_), which facilitate rapid iodide migration and lattice damage (Figure 1b) [10]. This defect evolution promotes further V_i_ formation (Circle I), while the resulting interstitial iodides and molecular iodine (I_2_) create an additional oxidation pathway, as I_2_ readily oxidizes Sn^2+^ to Sn^4+^ (Circle II). The generated Sn^4+^ does not remain as isolated point defects but further evolves into stable degradation products, most commonly forming SnO_2_ under illumination and trace oxygen. These irreversible oxidation end‐products accumulate at grain boundaries and buried interfaces, accelerating structural decomposition and contributing to long‐term device failure. This two‐stage defect‐driven pathway—ionic migration via V_I_ followed by I_2_‐mediated Sn^2+^ oxidation—represents a primary route for light‐induced degradation in Sn–Pb perovskites. A practical mitigation strategy is to suppress V_I_ formation, thereby slowing both ionic migration and I_2_ generation. For example, incorporation of octylammonium tetrafluoroborate (OABF_4_) into MA‐free Sn–Pb perovskites introduces BF_4_^−^ anions with strong binding affinity for Sn^2+^/Pb^2+^, thereby increasing the formation energy of V_I_ [10]. This reduces defect density and limits the production of interstitial iodides and I_2_ under illumination, enhancing photostability. Devices modified with OABF_4_ achieved a PCE of 23.7% and retained 88% of their initial efficiency after 1000 h of maximum‐power‐point tracking without cooling. These findings confirm that vacancy control via strong‐binding additives is an effective approach to simultaneously improve the efficiency and operational stability of Sn–Pb PSCs under light stress.

## 3 Crystallization Mismatch between Sn and Pb

In mixed Sn–Pb perovskite, the crystallization rates of the Sn‐ and Pb‐based components are often mismatched. Specifically, the SnI_2_–FAI complex exhibits a stronger binding energy (−0.92 eV) than the PbI_2_–FAI complex (−0.75 eV), indicating that Sn‐based intermediates nucleate more readily [18]. This disparity originates from the higher chemical reactivity of SnI_2_ toward organic halides compared to PbI_2_, which is attributed to the stronger Lewis acidity of Sn^2+^. As a result, Sn‐containing phases tend to nucleate and grow earlier during film formation. This nonuniform distribution produces rough surface morphology, elevated defect densities, and exaggerate phase separation, which together degrade the optoelectronic quality, efficiency, and long‐term stability of devices [25].

**FIGURE 4:**
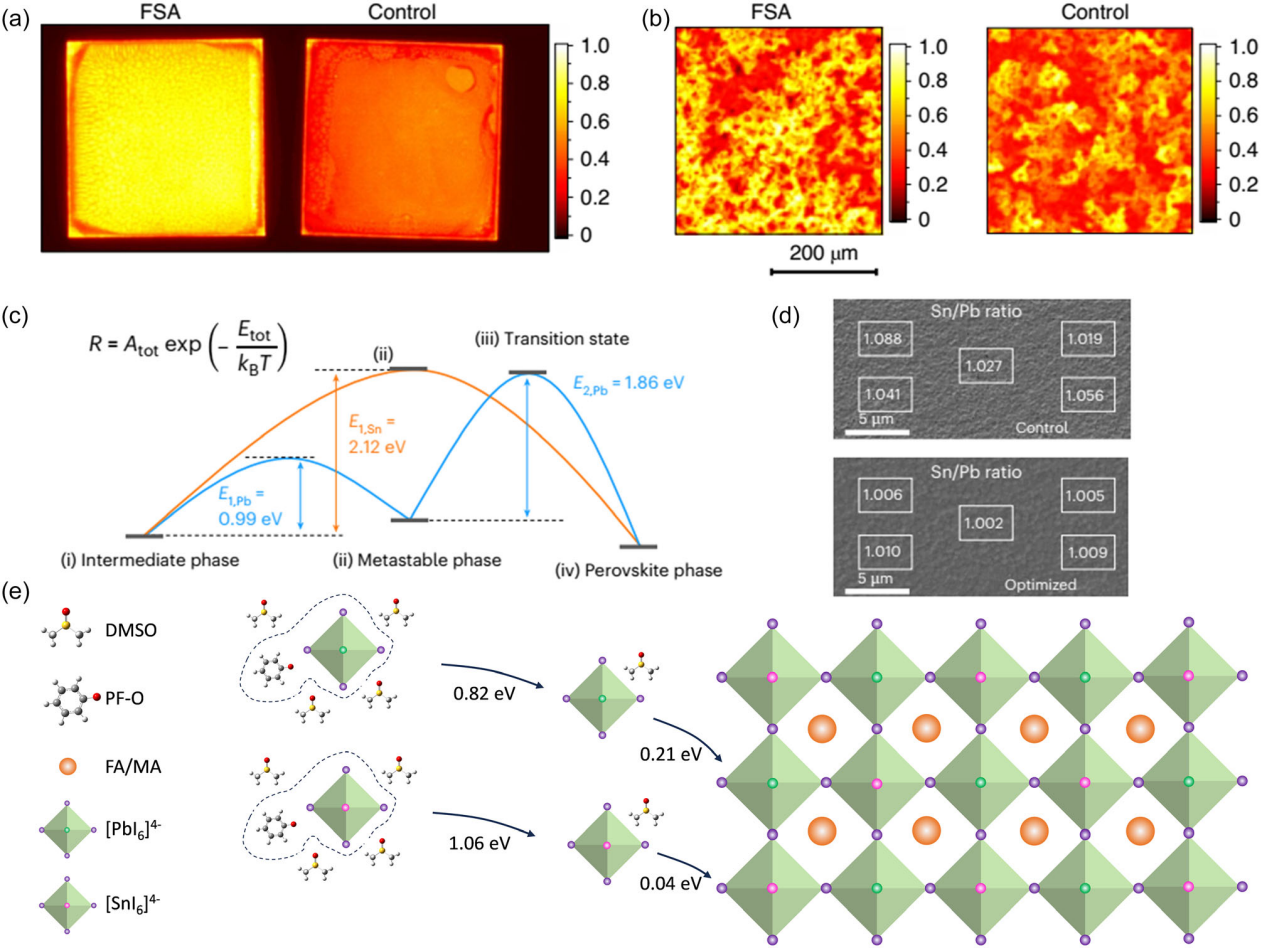
PL intensity imaging (a) and zoomed‐in micro‐PL mapping (b) of the control and FSA films deposited on glass substrates (size of 2.5 × 2.5 cm^2^). The color bars show the normalized PL intensity. The micro‐PL mapping area was 400 ×  400 μm^2^ with a step size of 5 μm and was selected from the substrate center. Reproduced with permission [26]. Copyright 2020, Springer Nature. (c) The desorption and transition barriers from the intermediate to perovskite phase. (d) The Sn/Pb ratio of the control and optimized perovskite films measured by energy‐dispersive spectroscopy. Reproduced with permission [27]. Copyright 2015, Springer Nature. (e) Schematic of PF–O^–^‐induced equilibrium crystallization in mixed Sn–Pb perovskite film. Reproduced under terms of the CC‐BY license [18]. Copyright 2025, John Wiley and Sons.

Lewis bases coordinating with Sn^2+^ have been widely used as an effective strategy to regulate the crystallization rate of mixed Sn–Pb perovskite film. For example, formamidine sulfinic acid (FSA) has been employed to improve the uniformity, electronic quality, and stability of mixed Pb–Sn perovskite films (Figure 4a,b) [26]. FSA coordinates with precursor constituents (PbI_2_/SnI_2_ and organic halides such as FAI/MAI) via dative bonding, in a manner similar to DMSO. Owing to its lower volatility compared with DMSO, the incorporation of FSA into the precursor solution enables delayed and more uniform crystallization. The zwitterionic tautomer of FSA can interact with both electron‐donating defects (e.g., FA/MA vacancies) and electron‐accepting defects (e.g., halide vacancies, undercoordinated Pb^2+^/Sn^2+^) on grain surfaces, providing defect passivation. Photoluminescence (PL) mapping confirms that FSA‐treated films exhibit improved spatial uniformity compared with the control. As a result, the PCE of small‐area devices increased from 19.4% to 21.7%, while large‐area (2.5 × 2.5 cm^2^) devices showed a substantial improvement from 13.9% to 17.5%.

Although the simple application of Lewis acid–base theory has achieved certain successes in regulating crystallization, in practice perovskite precursor solutions are multicomponent, complex systems whose reactivity is influenced by multiple factors. For instance, the crystallization behavior of Sn^2+^ and Pb^2+^ can be affected not only by their intrinsic Lewis acidity but also by parameters such as the number and strength of coordination bonds with solvents or additives [28, 29]. Addressing these complexities, Yang et al*.* systematically investigated the crystallization of mixed Sn–Pb perovskites from precursor solution to final film at the molecular level (Figure 4c,d) [27]. By dissecting four critical steps—precursor dissolution, intermediate‐phase formation via solvent extraction, coordinated‐solvent desorption, and metastable‐to‐final phase transition—they identified that the overall crystallization barrier is jointly determined by the desorption energy of DMSO from intermediate phases and the phase‐transition barrier. By optimizing the DMSO/[Pb+Sn] molar ratio, they achieved a uniform distribution of Pb and Sn across the film thickness, resulting in an efficiency improvement from 18.83% to 22.88%. This mechanistic understanding provides a more comprehensive framework for designing additive strategies that balance the crystallization rates of Sn‐ and Pb‐based components.

In Sn–Pb PSCs, the hygroscopic and acidic nature of PEDOT:PSS can accelerate Sn^2+^ oxidation and interfacial degradation, while its unfavorable work‐function alignment and suboptimal interfacial morphology further induce substantial nonradiative recombination losses. These limitations highlight the crucial role of HTL engineering in stabilizing mixed Sn–Pb PSCs [30]. In contrast to the precursor‐focused strategies described above, Wu et al. approached the crystallization mismatch and stability issues from the perspective of interfacial engineering, specifically by modifying the HTL (Figure 4e) [18]. Inspired by the antioxidative properties of polyphenolic compounds, they incorporated reductive phenol groups and strongly electronegative fluorine atoms into an organic conjugated framework, designing a multifunctional polymer (PF–OH) containing both units. Complementary DFT calculations showed that the binding energy difference between PbI_2_–DMSO and PbI_2_–FAI complexes is substantial (0.21 eV), whereas the difference for SnI_2_–DMSO versus SnI_2_–FAI is only 0.04 eV. This thermodynamic similarity explains why DMSO is ineffective at slowing the crystallization of Sn‐based perovskite film, thereby exacerbating the mismatch in mixed compositions. The strong binding affinity between Sn^2+^ and the phenol groups in PF–OH effectively modulated the crystallization and grain growth of Sn–Pb perovskite film, yielding films with fewer pinholes at the buried interface and longer carrier lifetimes. This interfacial modification boosted the PCE to 23.61% and substantially enhanced device operational stability. Furthermore, the generality of this approach was confirmed by extending phenol functionalization to a series of molecules, underscoring its potential as a universal strategy for improving the stability of Sn–Pb perovskite devices.

In addition to the binary SnI_2_–solvent and SnI_2_–FAI interactions, the presence of CsI—commonly incorporated in high‐performance Sn/Pb PSCs—further reshapes the precursor coordination environment, crystallization pathway, and stability [31, 32]. The introduction of Cs^+^ increases the Lewis acidity and bonding strength of the inorganic framework, thereby weakening the formation of unstable SnI_2_–DMSO adducts and shifting the equilibrium toward more robust inorganic–iodide coordination structures. This moderated coordination landscape reduces the intrinsic disparity between Sn‐ and Pb‐rich intermediates and slows the otherwise rapid nucleation of Sn‐based complexes, enabling more balanced crystallization kinetics. Recent studies additionally show that Cs^+^ participates directly in the intermediate phases (e.g., Cs–Sn–I frameworks), enhancing lattice rigidity and reducing fluctuation‐induced defects [33]. From a crystallographic standpoint, Cs^+^ promotes larger grain domains, reduces microstrain, and suppresses Sn/Pb compositional segregation. In terms of stability, CsI plays a multifaceted and synergistic role. Cs^+^ stabilizes the photoactive α‐phase, lowers halide vacancy concentrations, and suppresses light‐induced halide redistribution while simultaneously inhibiting I_2_ formation and slowing the Sn^2+^ → Sn^4+^ oxidation cascade. Moreover, Cs^+^ dampens ionic migration pathways by tightening the metal‐halide network and reducing the formation energy of V_i_, thereby mitigating both vacancy‐mediated degradation and Sn‐redox‐driven lattice collapse. Together, these effects establish CsI as a critical compositional modifier that not only reshapes the precursor complexation chemistry but also harmonizes crystallization kinetics and significantly strengthens the operational stability of mixed Sn–Pb PSCs.

Beyond achieving compositional uniformity, crystallization control in Sn–Pb perovskite films must also address crystal orientation. Seed‐induced crystallization has proven effective for producing uniform nucleation and orientation‐guided growth by lowering the critical Gibbs free energy for nucleation and accelerating the nucleation rate [10, 34, 35]. Chen et al. developed an in situ approach to generate an ABX_3_‐structured PbSnO_3_ seed layer within the precursor solution [36]. Owing to its strong interfacial binding and ≈98% lattice matching with the target Sn–Pb perovskite, this oxide‐based seed layer significantly lowered the nucleation barrier and induced highly oriented crystal growth. As a result, the seeded films exhibited markedly improved crystallinity, reduced residual stress, and fewer nonradiative recombination centers. Quantitatively, the incorporation of PbSnO_3_ seeds increased the carrier lifetime from ≈1139 to ≈1371 ns on PEDOT:PSS substrates and reduced the trap density (*N*_tr_ _ap_) from 4.23 × 10^15^ to 2.20 × 10^15^ cm^−3^, confirming substantial defect passivation. These improvements translated into device‐level gains, enabling single‐junction Sn–Pb solar cells to deliver a steady‐state PCE of 23.12% along with enhanced operational stability. This seed‐mediated crystallization strategy therefore provides a robust and quantifiable route to regulate crystallization kinetics and suppress deep‐level defects in mixed Sn–Pb PSCs.

SnF_2_ is now regarded as an essential additive for Sn‐rich and Sn–Pb PSCs because it suppresses the formation of Sn vacancies, lowers background p‐doping, and improves carrier lifetimes. Savill et al. [37] showed that, in FA/Cs‐based mixed Sn–Pb perovskite films, SnF_2_ reduces the hole density, mitigates Burstein–Moss shifts and lattice distortion, and thus decreases energetic disorder; however, excessive SnF_2_ leads to secondary phases and new nonradiative channels, highlighting its double‐edged nature. Chen et al. [38] systematically varied the SnF_2_ content in (FASnI_3_)_0.6_(MAPbI_3_)_0.4_ and found that an optimal ≈5 mol% SnF_2_ not only suppresses Sn^2+^ oxidation and Sn vacancies but also induces a “domain morphology” with columnar grains, yielding longer lifetimes and PCEs ≈20%; at higher loadings, F^−^ anions preferentially accumulate at the PEDOT:PSS/perovskite and perovskite/air interfaces, increasing interfacial defect densities and recombination. From a defect‐physics perspective, Treglia et al*.* [39] demonstrated that SnF_2_ reduces self‐p‐doping and passivates Sn^4+^‐related traps in FACsSnI_3_, especially at the surface where F^−^ strongly binds to Sn centers; yet Sn‐rich conditions with excess SnF_2_ promote Sn interstitials and iodine vacancies that open additional nonradiative decay pathways and limit radiative efficiency. Zong et al*.* [40] further showed that controlling the spatial distribution of SnF_2_ is as important as its concentration: using a Lewis adduct SnF_2_·3FACl, they drove SnF_2_ to form a continuous Sn(II)‐rich shell along grain boundaries in (FAPbI_3_)_0.7_(CsSnI_3_)_0.3_, simultaneously enlarging grains, suppressing Sn vacancies, reducing trap densities, and greatly improving resistance to moisture, heat, and light. Taken together, these studies indicate that SnF_2_ is an indispensable, but aggregation‐prone, additive whose content and spatial localization (bulk vs. interfaces vs. grain boundaries) must be carefully regulated: low‐to‐moderate loadings combined with controlled complexation or GB‐targeted delivery can maximize defect passivation and stability while avoiding F^−^ accumulation and SnF_2_‐rich defect clusters at critical interfaces.

## 4 Conclusion and Outlook

Mixed Sn–Pb perovskite films offer the optimal narrow bandgap for tandem integration and reduced lead content, making them a cornerstone material for high‐efficiency, next‐generation photovoltaics. However, as emphasized in the introduction, their stability and manufacturability remain constrained by two primary bottlenecks: the rapid oxidation of Sn^2+^ and the crystallization mismatch between Sn‐ and Pb‐based components. Over past few years, significant progress has been made on both fronts. Oxidation has been mitigated through TRPs, molecular antioxidants, phenol‐functionalized interfaces, and vacancy suppression. Crystallization mismatch has been addressed via Lewis base additives, solvent engineering, interfacial modulation, and seed‐mediated orientation control. These targeted strategies have yielded small‐area PCEs above 23%, operational lifetimes exceeding thousands of hours, and tandem devices with certified efficiencies beyond 30% [1]. Beyond oxidation and crystallization mismatch, the next generation of high‐efficiency (>24%) Sn–Pb PSCs will likely be limited by more complex, coupled degradation pathways, including ion migration, halide segregation under operational fields, and interfacial electronic/mechanical decay at buried contacts. Decoupling these intertwined processes will require operando multimodal characterization and defect‐specific passivation strategies capable of isolating individual degradation modes. Developing mechanically resilient interfaces and ion‐migration‐blocking interlayers will also be essential for pushing Sn–Pb PSC toward the 24%–25% efficiency regime with long‐term stability. The next phase of research should focus on integrated approaches that address multiple degradation pathways simultaneously—for example, combining vacancy suppression with oxygen adsorption inhibitors to disrupt both chemical oxidation cycles and light‐induced degradation loops. Deeper mechanistic understanding, enabled by in situ and operando characterizations, will be crucial to mapping the dynamic interplay between defect evolution, ion migration, and phase transitions. Furthermore, scalable manufacturing processes must be developed to maintain balanced Sn/Pb crystallization rates across large‐area modules. The insights gained from Sn–Pb PSCs are likely transferable to other NBG and lead‐free compositions, where similar oxidation and crystallization challenges exist. By uniting molecular‐level understanding with device‐level engineering, it should be possible to transform Sn–Pb PSCs from promising laboratory prototypes into commercially viable, stable, and environmentally responsible TSCs.

## Author Contributions

**Manman Hu**: visualization (lead), writing – original draft (lead). **Jianchang Wu:** writing – review and editing (lead). **Christoph Brabec**: funding acquisition (lead), writing – review and editing (equal).

## Funding

This study was supported by EnCN, Solar Factory of the Future, Solar Technologies go Hybrid, Deutsche Forschungsgemeinschaft (Grant SFB 953−No. 182849149), and Centro de Desenvolvimento de Materiais Funcionais (Grant 44‐6521a/20/4).

## Conflicts of Interest

The authors declare no conflicts of interest.
